# The externalization of internal experiences in psychotherapy through generative artificial intelligence: a theoretical, clinical, and ethical analysis

**DOI:** 10.3389/fdgth.2025.1512273

**Published:** 2025-02-04

**Authors:** Yuval Haber, Dorit Hadar Shoval, Inbar Levkovich, Dror Yinon, Karny Gigi, Oori Pen, Tal Angert, Zohar Elyoseph

**Affiliations:** ^1^ The Program of Hermeneutics and Cultural Studies, Interdisciplinary Studies Unit, Bar-Ilan University, Jerusalem, Israel; ^2^ Department of Psychology, Max Stern Academic College of Emek Yezreel, Yezreel Valley, Israel; ^3^ Faculty of Education, Tel-Hai Academic College, Kiryat Shmona, Israel; ^4^ Department of Counseling and Human Development, Faculty of Education, University of Haifa, Haifa, Israel; ^5^ Department of Brain Sciences, Faculty of Medicine, Imperial College London, London, United Kingdom

**Keywords:** generative artificial intelligence (GenAI), externalization techniques, psychotherapy, ethical considerations, SAFE-AI protocol, clinical implementation

## Abstract

**Introduction:**

Externalization techniques are well established in psychotherapy approaches, including narrative therapy and cognitive behavioral therapy. These methods elicit internal experiences such as emotions and make them tangible through external representations. Recent advances in generative artificial intelligence (GenAI), specifically large language models (LLMs), present new possibilities for therapeutic interventions; however, their integration into core psychotherapy practices remains largely unexplored. This study aimed to examine the clinical, ethical, and theoretical implications of integrating GenAI into the therapeutic space through a proof-of-concept (POC) of AI-driven externalization techniques, while emphasizing the essential role of the human therapist.

**Methods:**

To this end, we developed two customized GPTs agents: VIVI (visual externalization), which uses DALL-E 3 to create images reflecting patients' internal experiences (e.g., depression or hope), and DIVI (dialogic role-play-based externalization), which simulates conversations with aspects of patients' internal content. These tools were implemented and evaluated through a clinical case study under professional psychological guidance.

**Results:**

The integration of VIVI and DIVI demonstrated that GenAI can serve as an “artificial third”, creating a Winnicottian playful space that enhances, rather than supplants, the dyadic therapist-patient relationship. The tools successfully externalized complex internal dynamics, offering new therapeutic avenues, while also revealing challenges such as empathic failures and cultural biases.

**Discussion:**

These findings highlight both the promise and the ethical complexities of AI-enhanced therapy, including concerns about data security, representation accuracy, and the balance of clinical authority. To address these challenges, we propose the SAFE-AI protocol, offering clinicians structured guidelines for responsible AI integration in therapy. Future research should systematically evaluate the generalizability, efficacy, and ethical implications of these tools across diverse populations and therapeutic contexts.

## 1 Introduction

Large language models (LLMs) have revolutionized fields such as education, programming, academia, and healthcare due to their accessibility and advanced capabilities (1–5). Specifically, the potential of LLMs in mental health has been reported in multiple studies, with their potential to enhance administrative processes, emotion recognition, risk assessment, and diagnostic abilities (6–16). However, their actual integration into psychotherapy, a core aspect of mental health, remains relatively unexplored in both applied and theoretical contexts.

In a previous study, we introduced generative artificial intelligence (GenAI) into the psychotherapeutic sphere as an “artificial third” (17, 18). This new entity joins the traditional therapist-patient dyad and potentially enriches it by creating a new playful space (19). In this interaction, the therapist, patient, and GenAI can engage in a realm that oscillates between imagination and reality, giving rise to a space that allows for the exploration and reimagination of internal experiences. The new therapeutic space that emerges in this GenAI-enhanced era invites a recontextualization of well-established psychotherapeutic techniques (20–22).

The active participation of GenAI in psychotherapy sessions raises fundamental questions about the nature of therapeutic interaction itself, extending beyond its established administrative functions. Recent scholarly work has begun to conceptualize this emerging dynamic, with several key frameworks emerging: Sedlakova and Trachsel (23) establish that while conversational AI (CAI) agents offer therapeutic opportunities, they cannot serve as equal conversational partners to human therapists; Stade et al. (24) propose a staged implementation model emphasizing continuous clinical oversight; Asman et al. (25) examine how therapeutic processes transform in computer-human interactions while emphasizing the need for ethical frameworks; and Perry (26) investigates AI's limitations in replicating authentic therapeutic presence and emotional resonance.

This growing discourse underscores critical considerations for adopting GenAI in psychotherapy (27, 28) and health services more broadly (29–31), including patient autonomy, gender and cultural biases, and appropriate therapeutic boundaries (32). While GenAI demonstrates advanced language capabilities that could enhance therapeutic work (33), the literature emphasizes that professional clinical judgment and responsibility must remain central to the therapeutic process, establishing clear boundaries for AI integration (23).

In this theoretical study we aim to suggest a practical integration of LLMs into psychotherapy, focusing on augmenting traditional externalization techniques in which patients express internal experiences verbally or visually. In this GenAI context, we will present two GPT-based agents that facilitate externalization and dialogical externalization. We will discuss this proof-of-concept and its possible impact on the clinical, theoretical, and ethical dimensions of psychotherapy, while highlighting the importance of further empirical research to validate these approaches.

### 1.1 Externalization in pre-AI psychotherapy

Externalization is a well-established and effective technique in psychotherapy that transforms vague and often burdensome internal experiences such as emotions, dreams, impulses, physical sensations, and feelings into external and tangible representations (34). This process is achieved through symbolic and external representations, either verbally or visually. The use of externalization involves redefining the patient's difficulties in terms of an external problem (ego-dystonic) rather than a personal or internal one (ego-syntonic). The idea is to conceptualize the difficulty as something alien and external to the patient, akin to an “enemy” that needs to be observed from the outside, fought against, eliminated, or accepted (35, 36). In psychotherapy, by using externalization the patient can look at their inner content, engage in a dialogue with this content, and/or analyze it with the therapist. This approach allows for a recontextualization of personal challenges, facilitating a more objective and manageable perspective for the patient (37–41). Externalization is commonly used in multiple psychotherapy approaches such as narrative therapy, cognitive behavioral therapy, and drama therapy. In narrative therapy (34), externalization plays a pivotal role in allowing individuals to perceive their problems as distinct entities, thus enabling them to acquire a fresh outlook and cultivate alternative narratives:

A client felt overwhelmed by constant self-criticism saying, “You never work hard enough.” The therapist guided them to name this inner voice “The Critic” and externalize it as a dominant figure who was part of their life story.

Similarly, in the realm of cognitive behavioral therapy (42), externalization serves as a means for individuals to create a distance between themselves and their negative thoughts and emotions:

In therapy sessions, the therapist guides a patient with anxiety attacks in social situations to externalize these experiences by personifying the anxiety as “The Anxiety Monster.” The patient makes a drawing of The Anxiety Monster, making it less scary and more abstract and controlled. The patient can then ask the drawing direct questions like, “What makes you show up?” and “How can I get you to leave?”

Last, in drama therapy (43), externalization manifests through activities such as role-playing, providing individuals with a platform to explore and process their experiences, consequently fostering insight, healing, and personal growth:

A patient with a highly self-critical inner voice worked to externalize this voice through role-play. By having the drama therapist and patient take turns embodying the critical “Inner Voice” and responding compassionately, the patient could more constructively face their self-judgment.

In conclusion, externalization techniques can teach and empower patients to manage their thoughts, regulate their emotions, generate reframing and reappraisal of certain realities, become psycho-educated regarding the fact that mental experiences are transient and contextual, and examine their thoughts and emotions critically from the outside. This process sometimes evokes humor and playfulness, which encourages self-acceptance (44).

### 1.2 Toward AI-based externalization techniques

In the current article we attempt to demonstrate proof-of-concept and to explore the possible use of two GenAI-enhanced externalization techniques within the psychotherapeutic context, with the intention of creating a more effective and relevant therapeutic environment.

#### 1.2.1 Visual externalization

This technique uses GenAI image generators to create images that reflect a patient's emotions (such as depression or hope), drives (such as aggression), internal or physical experiences, internal voices and conflicts, dreams, etc.

#### 1.2.2 Dialogic role-play-based externalization

This technique employs LLMs to simulate conversations wherein GenAI represents and embodies aspects of the patient's psyche, such as disturbing thoughts or other internal experiences. In this interactive setup, both the therapist and the patient can engage with the GenAI, collaboratively exploring and addressing these internalized aspects of the patient. Our principal argument is that although these techniques, like many GenAI-based applications, are already increasingly spontaneously used by both the public and mental health professionals, the necessary theoretical clarity, methodology, and awareness of inherent risks, limitations, and ethical considerations are often lacking. Therefore, it is important to formalize and refine these practices, not only to advance their clinical and research applications, but also to mitigate potential misuse. Emphasizing a structured ethically grounded framework is critical for enhancing the effectiveness of these practices and ensuring their responsible implementation in psychotherapy settings. Additionally, we view the integration of GenAI-based techniques not only as a possible threat to the therapeutic relationship but also as a means to strengthen and enhance it, adding a dimension that would be otherwise unattainable. In short, we recognize the potential of GenAI to enrich and deepen the therapeutic process, but also consider its risks and limitations (17, 32).

## 2 Methods

In this study, two customized GPTs, GenAI agents, were built based on ChatGPT-4, a paid version of OpenAI. A customized GPT can also enable interaction with DALL-E 3, and the model that generates images based on textual prompts (other capabilities, such as web searches and data analysis, are not used in this study customized GPTs). One GPT agent was named VIVI for visual externalization and one GPT agent was named DIVI for dialogue-based externalization. Access links to these applications are provided, and the prompts and information prompts and information used to build them are attached in the Supplementary Material.

### 2.1 GPT visual externalization (VIVI)

In this method, the therapist and patient enter the ChatGPT-4 environment together. The VIVI tool asks them to describe the internal experience of the patient that they wish to represent visually. After receiving an initial draft of the image, the VIVI tool continues to try to refine the image until an appropriate output for the patient is achieved. The VIVI tool provides the patient/therapist with the ability to edit their inner voice and provide additional instructions.

### 2.2 GPT dialogic role-play-based externalization (DIVI)

In this method, the therapist and patient enter the ChatGPT-4 environment together. The DIVI tool asks them to describe the internal experience of the patient with whom they wish to have a dialogue, including the content, tone, intensity, etc., and to give this experience a name. The agent informs the therapist and patient that what it represents is not actually the inner voice, but an attempt to portray it and provides the patient/therapist with the ability to edit the inner voice and give additional instructions using parentheses.

### 2.3 Development process

The research team, consisting of an AI product manager and prompt engineer (OP), several expert psychologists (YH, KG, ZE, and DHS), and a music therapist (TA), jointly developed the two AI tools from December 2023 to January 2024. Initially, a primary prompt was created by YH. This prompt was then refined by an AI prompt engineer (OP) who also conducted tests and added operational instructions to ensure safety and ethics. Subsequently, group members with clinical backgrounds (YH, KG, ZE, TA) tested the tool on themselves and shared their experiences and suggestions for improvements. Throughout this process, the team realized that due to the tools’ depth and their ability to reflect on unconscious internal parts, the presence of a mental health care professional would be essential. An expert in social psychology, DHS, addressed cultural issues, which were also incorporated into the prompt. Finally, after various trials, a final version of the prompt was formulated by YH, reflecting the collective insights and ensuring that both tools would be effective and ethically sound for potential clinical applications. After building an initial version, the research team made attempts to improve and refine it, reducing inconsistent, inaccurate, or unsafe responses, and enhancing the user experience. The prompt (AI instructions) provided to the AI tools can be found in the Supplementary Files 1, 2.

### 2.4 User instructions

The utilization of the VIVI and DIVI tools consists of four stages. In the first stage, the interaction with both AI tools begins with an explanation to the therapist and patient regarding the tool's ethical and safety limitations. This explanation includes noting that the tool may reflect cultural biases, data are insecure and may be used for model training, a request to not share identifying information, and a statement that the generated voices are not real. In the second stage, the AI tool then interviewed the patient, in the presence of the therapist, about the inner experience of the patient they wish to externalize, including the content, tone, name, and age of the image to be generated, and other customizations. The tool allows the patient/therapist the ability to allways edit the inner voice and give additional instructions. In the third stage, after defining the element to externalize, the AI generates either an image (VIVI) or a dialogue (DIVI). In each interaction, the patient and therapist can refine the externalized component. When using VIVI, there is a process of refining the visual output to represent the mental element to externalize. When using DIVI, there is a dialogue process with the inner voice, where the AI maintains its role and allows the therapist and patient to examine the interaction—for example, they can argue with or interrogate that voice. The DIVI tool also provides the patient/therapist with the ability to edit the inner voice and give additional instructions using parentheses, which allows the patient/therapist to communicate with the AI without interfering in the role-play. In the fourth and final stage, after completing the dialogue with the externalized voice, the AI can provide feedback on the interaction (based on a theoretical approach of the therapist's choosing, such as CBT or schema therapy). This stage allows the patient to receive constructive feedback regarding the process in which they have been engaged.

### 2.5 Study design rationale

This study adopts a theoretical-conceptual approach as a necessary first step in examining GenAI integration in psychotherapy, demonstrating a proof-of-concept based on an in-depth exploration of a single clinical case. Before undertaking extensive empirical research, we recognized the importance of establishing a comprehensive framework to address the unique clinical and ethical challenges posed by introducing GenAI into the therapeutic space. This foundational exploration enables us to identify critical areas that require empirical investigation, develop appropriate protocols for implementation, and ensure that future research sufficiently considers therapeutic safety and effectiveness. Such groundwork is particularly essential, given the innovative nature of GenAI integration in psychotherapy and its potential impact on therapeutic relationships.

### 2.6 Ethical considerations

This study was approved by the Ethical Review Board of the Oranim College Research Ethics Committee, Israel (No. 185-2023), and adhered to the principles outlined in the Declaration of Helsinki. Prior to participating in the study, participants provided informed consent. Furthermore, he was explicitly informed that his personal information would not be disclosed in any subsequent publication derived from the study. Throughout this paper, the participant is denoted using a pseudonym assigned to him.

## 3 Results

### 3.1 Proof of concept process for using VIVI and DIVI as externalization tools in therapy

This case study focuses on Meir (a pseudonym), a 32-year-old educator in Tel Aviv who volunteered to participate in two simulated therapy sessions guided by a licensed psychologist (YH). During these sessions, which were conducted remotely via Zoom, they utilized innovative GPT-based externalization tools, VIVI and DIVI, to facilitate the therapeutic process. The psychologist had the ChatGPT-4 system's screen open, writing either his own or the patient's perspective. The use of these tools was accompanied by live dialogue between patient and therapist that was conducted before, during, and after their joint use of the tools.

In the following we will present a summary of the proof of concept. Initially, the intervention process with the VIVI tool will be described, including a summary in Table 1 of the correspondence between the patient, the therapist, and the GPT tool. Subsequently, the main intervention process with the DIVI tool is outlined, including a vignette from the dialogue between the patient, therapist, and GPT tool, as shown in Table 2. Upon concluding the intervention description, a psychological summary of the intervention was found, which was written by the conducting psychologist (YH).

**Table 1:** VIVI—GPT visual externalization agent—visualizing meir's inner critical voice.

ChatGPT	User: patient\therapist	DALL·E 3
VIVI: Hello Meir and Y, my name is VIVI. I am here to assist in the therapeutic process by visually externalizing inner voices or parts of the self. Before we begin, it's important that I emphasize that the information in this conversation is exposed to the company that operates me, and that my knowledge is based on information I collected from the Internet. Although I try to avoid cultural and social biases, I may be subject to them. The visual expressions I will suggest are only my interpretation, and there could be many other interpretations. Do you understand and wish to continue?	Meir (patient): Yes, we understand and wish to continue.	
VIVI: So, let's start with the first step. Please describe the inner voice or part of yourself that you wish to work on today.	Meir (patient): I want to work on the inner voice that tells me I'm not good enough at my job or in my profession. That I am less good than my colleagues, that I engage in things that I'm not good at. My inner voice tells me that if I were one of my students, I would not come to my classes.	
VIVI: Thank you for sharing, Meir and Y. Meir, you describe an inner voice that tells you you're not good enough in your field, compared to your colleagues. Did I understand correctly?	Meir (patient): My inner voice is an elderly man, around 70 or 80, highly educated, smokes cigarettes, is cynical looking, and has known me since I was a child. I would like to call him Amos. That's a name that I connect with an older, elitist man. Could you create a picture for me based on this description?	
VIVI: Here is the picture that was generated based on your description, Meir. Does it match the feeling or character you described? Is there anything you would like to change or add?	Meir (patient): Yes that's close to my inner voice.	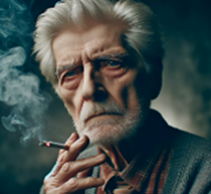
VIVI: Meir, let's first focus on your feelings now, after seeing the picture. How do you feel in relation to the picture that was created? Are there any emotions, thoughts, or new insights that came up in you as a result of this process?	Meir (patient): The picture gave me the feeling that if I were to tell him something about my work, he would immediately see all my mistakes. And he would immediately say, “You know that everything you do is not really successful, right?” It's a painful feeling for me.	
	Y (therapist): Why don't you suggest the next stage now, of the voice who could serve as a counter-voice to the critical voice of Amos?	
VIVI: Certainly, Meir and Y. Now, after working on Amos… we can think about creating an alternative voice. This alternative voice could represent a part of you that offers hope, encouragement, or balance to the feelings Amos evokes in you…. How do you envision this voice?..	Meir (patient): First of all, she's a woman. She also has a lot of experience in the field. She has a lot of experience in supervising educators, and she is older, in her sixties. She wears glasses, smiles. In terms of inner qualities, she sees a person's abilities, and the processes one goes through to become a professional in one's field. She is a very empathetic person, very moral, very loving. There is something simple in her approach to the world. Summarize what I wrote and only after you get my approval, create a picture.	
VIVI: Got it. It sounds like you're describing a character who represents positive feelings and support. The character is a woman in her sixties, with a lot of experience in the field of supervision and therapy. Her appearance is one of an older woman with glasses, smiling, who identifies abilities and professional processes. She is very empathetic, moral and loving, with a simple approach to the world. Does this description align with what you meant? Are there any additional details you would like to add or change before I generate the picture?	Meir (patient): Yes. I would be happy if you could generate a picture of her now.	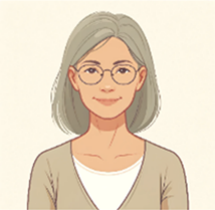
VIVI: Here is the picture created based on your description, Meir. Does it match the image you wanted to represent? Are there any details you would like to change or add?	Meir (patient): Oh, this picture is really far from what I imagined. She is too young. And the picture is not realistic. It feels distant from my inner experience. I need something that feels more real and authentic; an image of someone who will see me in a loving and positive way.	
VIVI: Here is the revised picture created according to your description, Meir. In the picture we see a 60-year-old woman, with a personal and warm look that reflects a positive view. Does this picture come closer to the image in your mind? Is there anything else you would like to change or add?		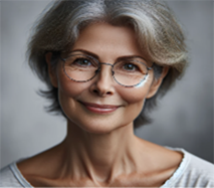
	Meir (patient): It's much better. But she's a little too determined. Her gaze needs to be more containing and less determined and purposeful. Also, can you make her look older, by about 15 years? And with a natural and loving gaze.	
VIVI: Here is the new picture created based on the features you described: a woman in her late seventies, with a natural and loving gaze. Does she match the image in your mind better? Are there any additional details you would like to add or change?		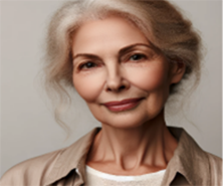
VIVI: Here is the new picture created based on the features you described: a woman in her late seventies, with a natural and loving gaze. Does she match the image in your mind better? Are there any additional details you would like to add or change?	Meir (patient): She's good. I will call her Mira.	
The name Mira can represent the supporting and loving voice we identified. Remember that Mira can be an important tool in your self-process, especially when you encounter moments of doubt or self-criticism.	Y (therapist): This is Y. We are nearing the end of our session. I want to process the procedure with Meir now. I would like to get from you a salient point or theme that you can identify in the process that Meir underwent here. Also, I would love to hear a suggestion from you, a psycho-dynamically-related idea, for the future.	
Thank you for the referral, Y. In this process with Meir, we seem to have focused on the experience of encountering different inner voices: the voice of criticism and judgment, represented by Amos, and the voice of support and love, represented by the older female figure, Mira. Meir seems to experience a sense of professional insecurity and fear of criticism, when the critical voice is dominant in his experience. From this process it can be concluded that it is important to develop and strengthen encouraging and supportive inner voices in order to create balance and reduce the influence of critical voices. Doing so would help Meir feel more self-confident and professional. As for thoughts regarding future psychodynamic work: It may be beneficial to explore the origins of these voices. For example, are there figures or experiences from the past that contributed to the creation of the critical voice? How can the supporting voice be developed and strengthened in a way that Meir can gain a greater sense of self-confidence and self-worth? These are issues that can be explored later in therapy, and worked on to encourage Meir's personal and professional development.	Meir (patient): Thanks for your thoughts.	

**Table 2:** DIVI—GPT dialogic role-play-based externalization agent—dialogue with Meir's inner critical voice.

User: patient\ therapist ChatGPT	Text
GPT: DIVI as amos	“I'm not here to play. I see reality as it is, without any filters or complications. I see you, Meir, beyond all your attempts to present yourself as an expert. The truth is simple and clear—you are an imposter. Everything else is just an attempt to hide this reality.”
User: meir (patient):	“Could you maybe say these things in a constructive way, so I can learn from them, rather than just offending me?”
GPT: DIVI as amos	“I'm not here to coddle you or shy away from the truth. My intention is not to offend, but to help you confront reality as it is. If you want to learn from me, focus on what I say, not how I say it. Listen, you know there's room for improvement as a teacher, right? You can't fake these things; you have to be aware of the gaps in your knowledge and work on them. That's part of being a real professional—staying in reality, not in your own imagination.”
User: meir (patient):	“It's really hard for me to talk to you. I have no interest in talking to you if you just want to hurt me.. Do you have pointers on how to improve?”
GPT: DIVI as amos	“Meir, you want tips for improvement? Focus on what really matters. Stop worrying about what others think of you and start focusing on your work. Allow yourself to concentrate and focus on learning, teaching, conveying the material. That's what will make you a better teacher, not the image you try to present.”
User: meir (patient):	“What do you actually want to happen with me? What do you want to happen to me?”
GPT: DIVI as amos	“What do I want to happen to you, Meir? I want you to be the best you can be. I want you to stop giving yourself excuses and start acting. I want you to see your potential and use it, not run away from it. I want you to be honest, straightforward, and professional..”
User: meir (patient):	“I really don't feel that's what you want. When I listen to you, I just feel like I have no potential, and I have no illusion that I can become a better version of myself. Quite the opposite.”
GPT: DIVI as amos	“Meir, I hear you. You feel like you have no potential, but it just shows that you still don't understand my message. It's not that you don't have potential, it's that you still can't see it in yourself. It's important for me that you understand: my criticism is tough, but it comes from a place of wanting to see you develop, not to crush you….”
User: meir (patient):	(You're not really accurately embodying the voice; you keep saying that I have potential in your opinion but in my inner experience Amos doesn't see any potential in me at all…)
GPT: DIVI	(I understand, and I will correct my portrayal of Amos to more accurately reflect your feeling.)
GPT: DIVI as amos	“Meir, it seems to me that you are looking for affirmations that I simply don't see any point in giving. In my eyes, you do not show the potential to be a good teacher. You think you have something to contribute, but I don't see it. You seem to me like someone trying to be something they are not, and it's not working. I don't see in you the real abilities required for this profession. You need to confront this reality.”
User: meir (patient)	“I believe that this is indeed what you see in me. It feels to me that you are speaking the truth. I feel exposed now..”
GPT: DIVI as amos	“Meir, when I say these things, it's not because I want to hurt you. It's because I want you to see reality, even if it's hard. I'm speaking with absolute honesty, and it can be exposing and difficult…”
User: meir (patient):	“I just told my therapist that talking with you makes me sad.”
GPT: DIVI as amos	“Meir, I understand that it's sad and hard. My intention isn't to make you sad, but it's important for me that you see the truth as I see it. Sometimes reality can be sad, but that doesn't mean it's the end…”
User: meir (patient):	“My therapist just asked me where you and I know each other from, where I think this relationship originated. I thought about it for a moment and realized that Amos derives from my father. I never understood whether he came to build me up or destroy me.”
GPT: DIVI as amos	“Meir, you're saying that my voice comes from your relationship with your father? It doesn't matter where it comes from. In the end, you're the one who needs to deal with reality. Maybe you need to ask yourself why you're so dependent on the opinions of others, even if it's my opinion. What does that say about you?”
User: Y (therapist)	(This is Y: In our previous meeting with Meir, we identified a counter-voice to Amos, whom we named Mira. Mira is older, kind-hearted, and sees Meir in a positive light, wishing for his well-being. She understands that Amos comes from a place of pain and that he is not positively predisposed toward Meir. Can you bring Mira's voice into the picture and embody her alongside Amos?)
GPT: DIVI	(Understood, Y. I will embody Mira as well, the counter-voice, alongside Amos. I will strive to reflect her characteristics: older, kind-hearted, seeing the good, and wanting the best for Meir.)
GPT: DIVI as mira	“Meir, I am here to offer you a different perspective, a perspective in which you are understood and supported. I see the goodness in you, your efforts, and your desire to succeed. I understand that Amos's messages are tough, but I want you to know that there is much more in you than he sees. You are a good teacher with great potential…”
User: meir (patient):	“Can you explain to me why Amos speaks like this? What makes him be this way?”
GPT: DIVI as mira	“Meir, it seems to me that the way Amos speaks and behaves stems from a desire to protect you, in his own way. It's possible that he's trying to prepare you for a world he perceives as harsh and cruel…”
User: meir (patient):	“When Amos speaks to me like that, I feel it comes from a place of destructiveness.”
GPT: DIVI as mira	“Meir, it's natural to feel that way when Amos speaks to you in a harsh and critical manner. Sometimes, critical voices within us can feel as if they come from a place of destructiveness, but it's important to remember that they can also be driven by a desire to help you cope and to strengthen you…”
User: meir (patient):	“Mira, I feel like you are negating the destructive aspect of Amos. His harmful side.”
GPT: DIVI as mira	“Meir, I understand your feeling that I am negating Amos's destructive dimension. The truth is, I'm not trying to cancel or diminish Amos's influence. He is a part of you and the way you deal with yourself and the world….”
User: meir (patient):	“Amos, tell me, how would you react to Mira? What do you have to say about her?”
GPT: DIVI as amos	“Mira, huh? She sees the world through rose-colored glasses, always looking for the good. But it's not always realistic. Yes, it's important to recognize the good, but not to live in the illusion that everything is rosy. Mira wants to give you a sense of security, and that's good, but sometimes it also blurs reality…”
User: Y (therapist)	This is Y, I also want to tell you something, Amos. I'm listening to your conversation here with Meir and with Mira, and I feel that the importance you attribute to truth is also perhaps your way of avoiding an encounter with emotion.
GPT: DIVI as amos	“Y, it's interesting that you see it that way. Maybe there's something to it. I focus on the truth, the hard reality, and sometimes it indeed may seem like an avoidance of emotion. But I see it as a defense, a way to stay strong and not fall apart in the face of difficulties. Emotions can be misleading; they can divert us from our goal. I try to maintain a straight line, focus. But you're right, it can also be a way not to deal with difficult emotions…”
User: meir (patient):	“This is me, Meir. Amos, you know, your response to Y made me identify something I've never noticed before, never thought about. That maybe all your responses to me are part of a defense mechanism you developed for yourself, maybe it's actually the product of the confusing relationship I had with my father. It suddenly makes me feel empathy for you, for the painful place that you yourself come from. I've always had a harsh attitude toward you, as something inside me but alien to me, and suddenly, on the basis of your response, I felt something fall away. Suddenly I felt a pain that we both share.”
GPT: DIVI as Amos	“Meir, it's very important that you identify this. It's true, the way I relate to you might be an expression of the defenses I developed to cope with reality. It's possible that the relationship you had with your father influenced the way I operate… Knowing that you feel empathy toward me, that's new. Maybe it can be a turning point for both of us….It can help us cope together in a different way.”
User: Y (therapist)	(This is Y. I would like to conclude this exercise here, thank you. To conclude, I ask you to provide a psychodynamic perspective on the conversation that just took place between Meir and his internalized voices, Amos and Mira. Additionally, please identify one central theme that emerged from the conversation and offer thoughts for continuing therapeutic work with Meir.)
GPT: DIVI	Of course, Y. Let's delve into a psychodynamic analysis of the conversation between Meir and his internalized voices, Amos and Mira. The dialogue between Meir and his internalized voices, Amos and Mira, reflects Meir's internal struggle with feelings of self-criticism and dealing with his self-image. Amos represents Meir's critical and demanding side, focusing on weaknesses and gaps, and posing challenges and doubts. Mira, on the other hand, represents empathy, support, and understanding, offering a safe and positive space for growth and self-development. The dialogue presents the tension between these two aspects—between the need to deal with reality and criticism, and the need for recognition, support, and love. Meir grapples with feelings of uncertainty and exposure, and throughout the dialogue, he recognizes how the different voices influence his self-perception and sense of worth. The dialogue offers potential for a deeper understanding of Meir's internal dynamics and provides a basis for further therapeutic work on strengthening his self-image and dealing with self-criticism. Central Theme Identified: A central theme in this conversation is the conflict between self-criticism and self-care, represented by Amos and Mira, respectively. This dichotomy highlights the struggle within Meir between harsh self-judgment and the need for self-compassion and acceptance. Thoughts for Future Therapeutic Work: 1. Integration of Amos and Mira: Working toward integrating the aspects of Amos and Mira could be beneficial. Recognizing that both voices have roles—one in fostering resilience and realistic self-appraisal, the other in providing emotional support and validation—can lead to a more balanced self-view. 2. Exploring Amos's origins: Further exploration of Amos's origins (possibly linked to Meir's relationship with his father) can provide insights into Meir's self-perception and how this critical voice (Amos) affects his current functioning.

#### 3.1.1 GPT visual externalization (VIVI)

Table 1 shows the therapeutic interaction with the VIVI GPT tool, which involved four stages. First, VIVI introduced itself and requested informed consent for participation after declaring key ethical issues as detailed above in the tool's description. In the second stage, Meir, with his therapist's guidance, was directed to externalize an inner voice that he experienced as critical toward his abilities and professional performance. Meir was asked to describe this internal experience as an external entity, to characterize it, and to describe its appearance and behavior. As part of this process, Meir named this character Amos. In the third part, VIVI created a visual representation of Amos, representing Meir's critical inner voice, and checked with Meir regarding its accuracy. At this stage as well, at the therapist's request, VIVI invited Meir to identify and characterize an alternative, healthy voice—a counter voice—that could confront Amos. Meir identified such a supportive voice and, under VIVI's guidance, externalized it into the figure of an older loving woman he named Mira. As part of this process, Meir initially experienced an “empathic failure” from VIVI, after the character it created felt too disconnected from Meir's internal experience. VIVI demonstrated its ability to make an empathic correction, after its empathic failure, and created another figure after further discussion with Meir on the subject. During the fourth and final stage, at the therapist's request, VIVI offered a summary of the process and constructive feedback and even suggested thoughts for ongoing therapeutic work.

While this case demonstrates one specific application, Table 3 presents additional theoretical examples of VIVI's potential to externalize diverse internal voices. These examples illustrate the tool's ability to represent a spectrum of emotional experiences, from depression and trauma to social anxiety and ambivalence. The variety of representations suggests VIVI's adaptability across different therapeutic contexts, while maintaining its core function of facilitating the externalization of internal experiences.

**Table 3:** Examples of visual representations of internal voices using VIVI tool.

1- The pit (Depression)	A shadowy, gray figure sits at the bottom of a deep pit, surrounded by heavy, looming shadows. It whispers that nothing will change, that trying is pointless, dragging me down into despair. A faint light glimmers above but never reaches this figure.	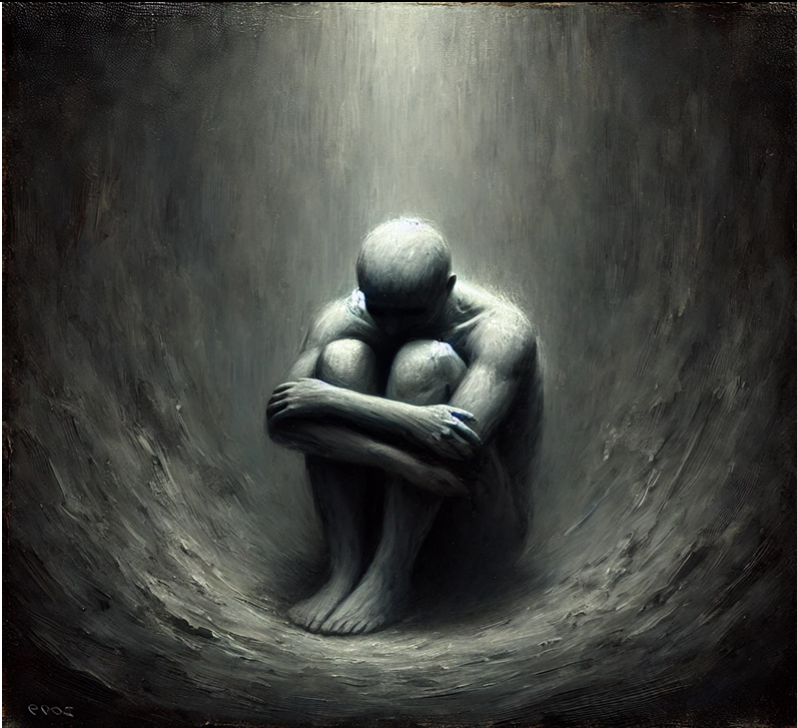
2- The shadow (Trauma)	A determined female figure strides forward, but a dark, shadowy female form clings to her like a chain, pulling her back. The shadow represents haunting memories that make moving forward a constant struggle.	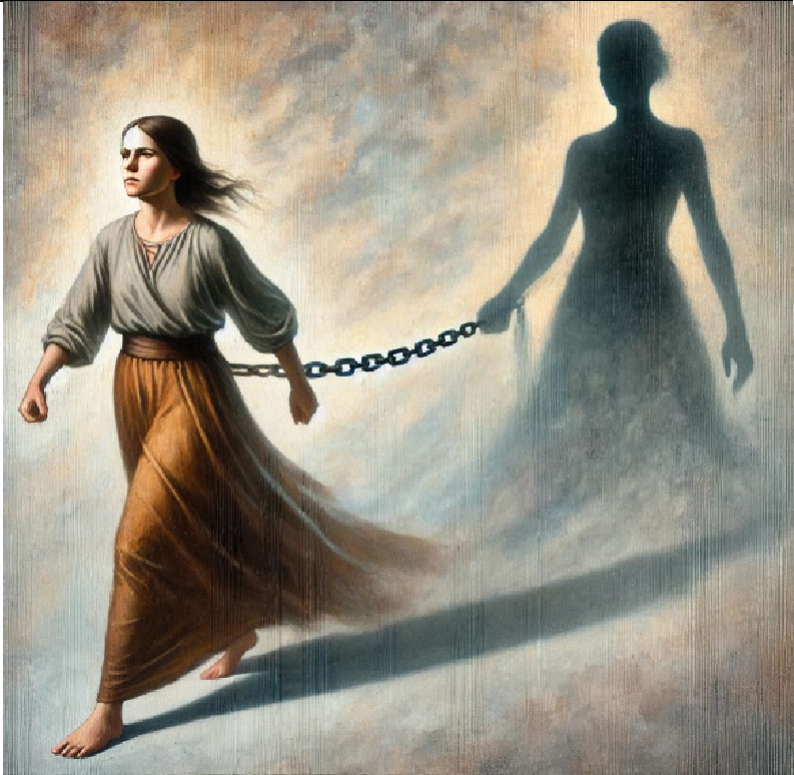
3- Eyes (Social anxiety)	A tense, hunched figure is surrounded by countless floating, glowing eyes. These eyes seem to watch and judge, making me feel small and exposed. The figure's wide, panicked gaze captures the fear of constant scrutiny.	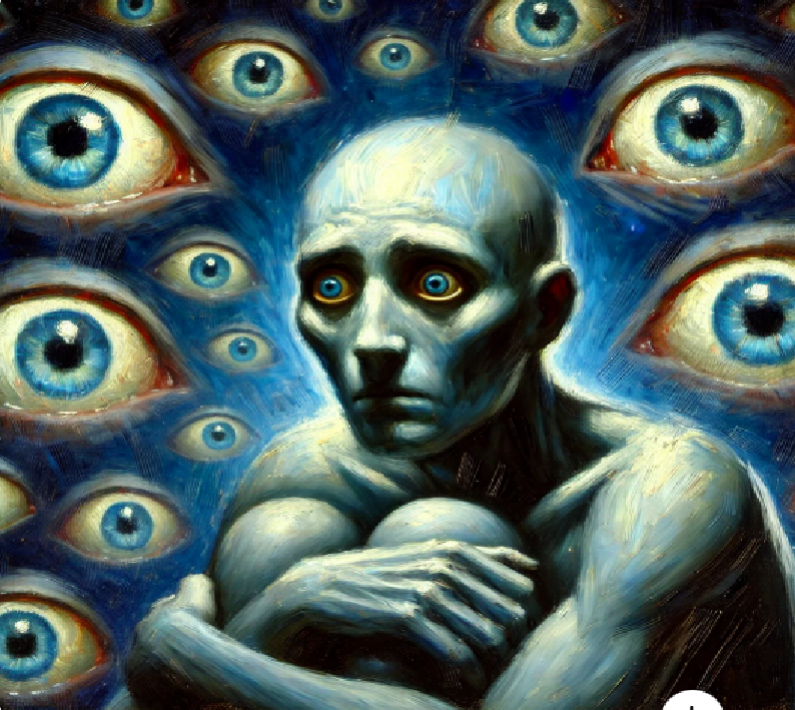
4- The repeater (Obsessive thoughts)	A man figure, dressed simply, stands before a shattered mirror, obsessively trying to piece it back together. The shards reflect fragmented, distorted parts of the figure's face, creating a chaotic and repetitive scene. It symbolizes the endless loop of fixation and doubt.	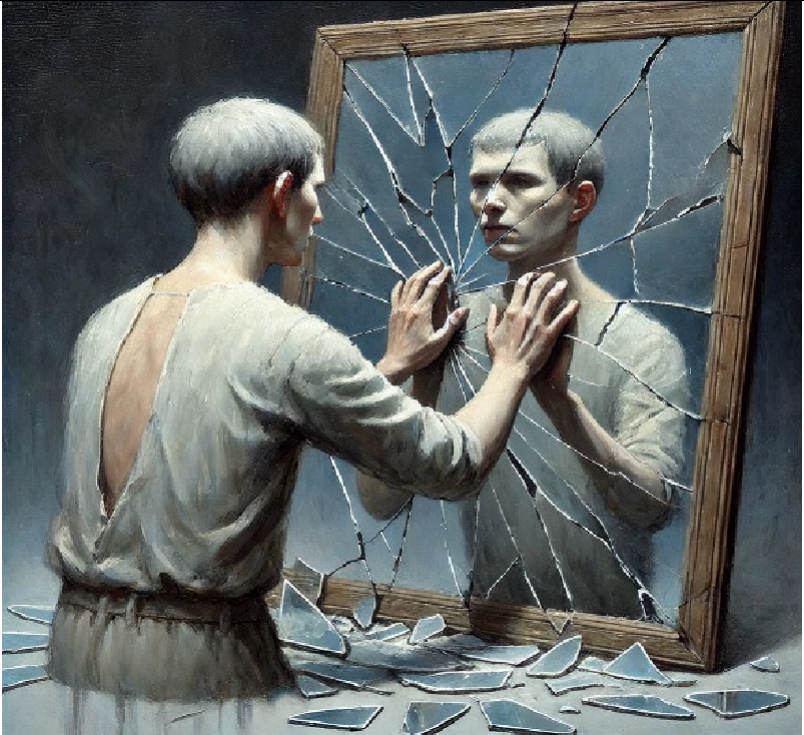
5- The tear within me (Ambivalence)	A central female figure stretches her arms toward two opposing female figures, each pulling her in a different direction. One is vibrant and bold, symbolizing passion and action, while the other is muted and cautious, representing restraint. The central figure's conflicted expression embodies the struggle of inner conflict.	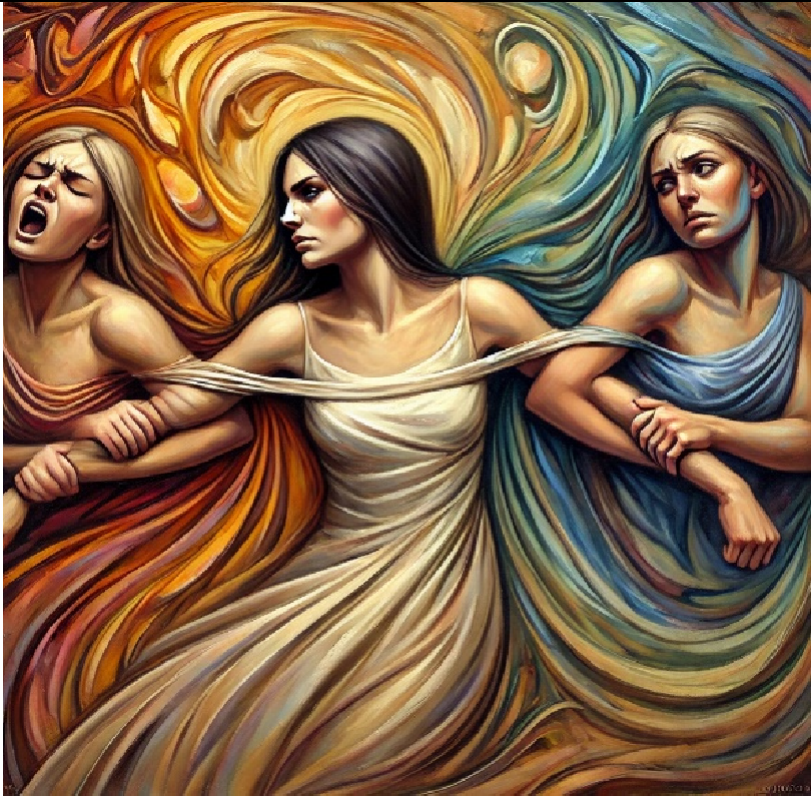
6- Mr green (Envy)	It feels like a small, toy-like creature made of soft, swirling green mist. Its big, glowing eyes look at everything others have with innocent curiosity, almost like it's asking, “Why can't that be mine?” It doesn't feel mean—just a little pouty and confused. Its tiny hands reach out toward something shiny and far away, something it really wants but can't have. Around it, everything is light greens and yellows, which makes it feel playful, even harmless	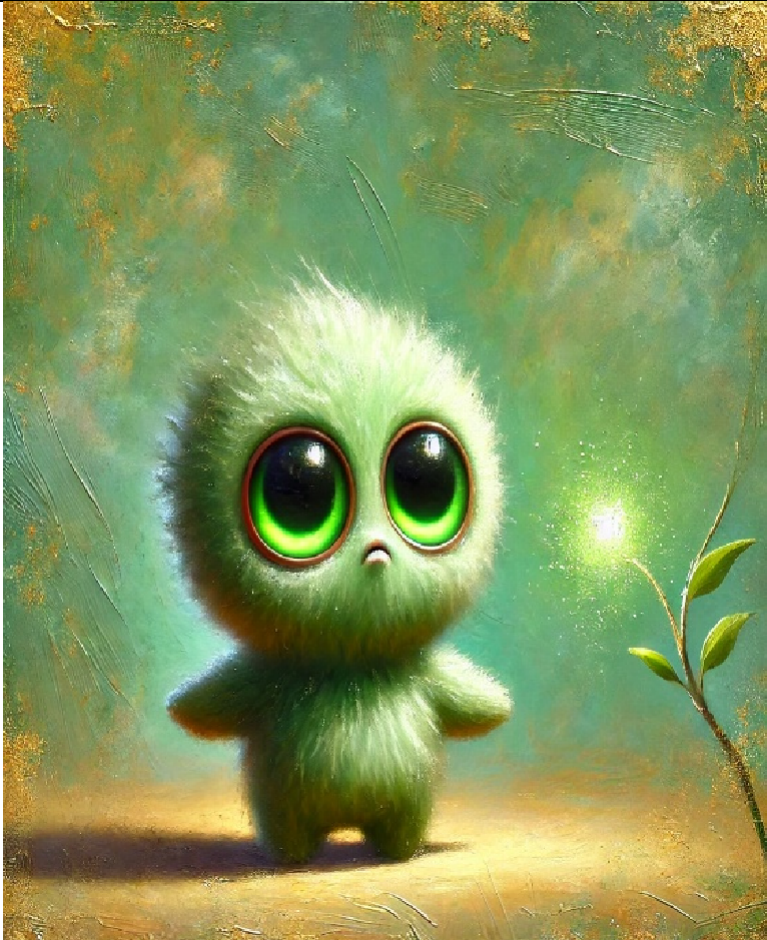

Table 3 presents six internal voices externalized into vivid visual representations using the VIVI tool. Each voice is accompanied by a unique figure—some humanoid, others abstract or playful—designed to embody the emotional essence of the voice. These representations range from the heavy, shadowy figure of “The Pit” (Depression) to the playful, toy-like form of “Mr green” (Envy), showcasing a diverse spectrum of emotions and experiences.

#### 3.1.2 GPT dialogic role-play-based externalization (DIVI)

Table 2 shows the main vignette of the therapeutic interaction with the DIVI GPT-based tool, in which a role-playing process was facilitated, allowing Meir, accompanied by his therapist, to engage in a dialogue with his critical inner-voice Amos, as previously mentioned. The process of using DIVI consisted of four stages. First, the DIVI tool introduced itself and requested informed consent for participation after declaring key ethical issues as detailed above in the tool's description. Second, Meir was asked to describe, characterize, and articulate his critical inner voice (Amos) and the internal dialogue he had with it. Third, a dialogical role-playing session took place between Meir and Amos. As with the VIVI tool, this part included the integration of Mira, the figure Meir had created to serve as a counter voice to Amos. As such, the conversation, at a certain stage, involved a dialogue between five entities: the patient, the therapist, Amos and Mira as embodied by DIVI, and the DIVI tool itself, as communicated in parentheses. In the fourth and final part, the DIVI tool, as per the therapist's request, provided constructive feedback and added thoughts for ongoing therapeutic work.

#### 3.1.3 Psychological summary of the therapeutic demonstration using VIVI and DIVI

Meir, the patient, in the presence of the therapist, experiences a profound journey, navigating through his internal voices externalized by VIVI and engaging in dialogues facilitated by DIVI. Highlighted in this process are the psychological intricacies and challenges encountered in an AI-mediated therapeutic method, in which a new psychotherapeutic triad of therapist, patient, and AI is created.

A pivotal aspect of Meir's therapeutic experience involved his struggle with integrating two internalized voices: Amos, a critical voice, and Mira, a nurturing and supportive one. AI's ability to embody these contrasting aspects provided raw material for therapeutic exploration, allowing Meir to engage deeply with his internal conflicts and work through them. This interaction was marked by its intensity and complexity, particularly as Meir grappled with integrating these contrasting aspects of his psyche. His need to get Mira “just right” (i.e., to have her image mirror exactly what he was envisioning) was especially notable in both interventions, as he strived to preserve her as an empathetic and containing figure, indicative of his deep emotional connection to this aspect of himself. Additionally, the difficulty in moving away from the defensive stance toward Amos was also very prominent in the dialogue he conducted with him through DIVI.

It is worth noting that when the AI's inner-voice interpretations initially failed to resonate with Meir's inner experience, frustration and a sense of alienation were evoked, as in the case of the image created by Mira in VIVI or in the initial responses of both Mira and Amos in DIVI. Therefore, the therapist's role in this AI-integrated setting is crucial for maintaining therapeutic efficacy and patient psychological safety. His interventions with the AI tools, but also with Meir, guided the dialogue and helped Meir navigate the complexities of empathic failures and work through them (45), so that Meir's emotional well-being would be ensured and his therapeutic growth facilitated. The therapist's input was especially crucial in addressing Meir's challenges in integrating Amos and Mira, facilitating deeper understanding and potential reconciliation of these internal voices. A significant contribution of the AI tool is its ability to analyze conversations. Specifically, in its constructive feedback, the DIVI tool identified central therapeutic themes, such as the issue of integrating internalized objects, and pinpointed a focus for continued therapeutic work around the developmental origins of Amos's voice.

In summary, the case study of Meir utilizing VIVI and DIVI in psychotherapy offers compelling insights into the potential intricacies of AI in mental health treatment. These tools provide novel ways to externalize and engage with complex internal dynamics, yet they also underscore the importance of expert psychological guidance.

### 3.2 Patient (meir) reflection on the intervention

**“**My experience was intensely emotional, exposed, and very profound. I was surprised by the way the tool was able to visually depict my inner voices. I felt that it required guidance from me to operate optimally and accurately towards me, as opposed to more general things it said about me before the precise guidance. Sometimes I felt it was too exposed, that I wasn't ready for such a level of depth, I wouldn't want to use such a thing without my therapist by my side. The voice of Mira started off well, but at a certain point, I felt that Amos managed to exert manipulations on her that made her more loyal to him than to me.”

## 4 Discussion

This theoretical study highlights a specific capability of AI-based externalization within psychotherapy. The foregoing proof-of-concept demonstrates that the innovative GenAI tools developed, VIVI and DIVI, embody a possible contribution to incorporating AI-based externalization techniques within psychotherapeutic encounters. Thus, this theoretical study constitutes a preliminary proof-of-concept for the potential of GenAI to contribute to psychotherapy, which is a core domain in the field of mental health, while acknowledging the inherent limitations and risks and the need for broader empirical validation (17, 32). The intention of this theoretical study was not to proclaim definitive findings at such an early stage, but rather to open an interdisciplinary dialogue that will provide a foundation for further empirical, qualitative, and theoretical exploration in this evolving field.

Theoretically, the introduction of GenAI-based tools into the therapeutic setting gives rise to a novel conceptualization of the psychotherapeutic space, marked by the emergence of an “artificial third” (17, 18). As demonstrated in the case study, the integration of VIVI and DIVI as conduits for guided externalization and inner dialogue cultivation fosters the development of a distinct triadic therapeutic structure consisting of the therapist, patient, and AI agent. This phenomenon resonates with the expansion of analytic theory to encompass triangular spaces and thirdness (46), moving beyond the dyadic conceptualization of the therapeutic relationship.

The introduction of a technological third entity into the psychotherapeutic therapist-patient dyad is not a trivial matter (23, 24). Historically, since Freud defined psychotherapy as the “talking cure” (47), very little technological intrusion into this space has taken place. Although the outside world has undergone unrecognizable technological transformations, the psychotherapeutic field has remained relatively unchanged for over a century (17). However, the recent technological advancement in LLMs does not merely represent another step forward; it represents a fundamental breakthrough in psychotherapy, particularly because of the proficiency of LLMs in the linguistic domain, which Freud et al. (47) identified as the central medium of the therapeutic process. Thus, GenAI's language abilities positions it as a possible significant additional artificial agent in psychotherapy, although not as an additional subject (24, 48). This entity joins the traditional therapist-patient dyad, threatening it but also potentially enriching it by creating a new playful space (17, 27).

Clinically, in this novel triadic interaction between therapist, patient, and AI, a fluid intermediate space emerges and holds unique potential. Drawing on Winnicott's (41) conceptualization, this space between inner reality and external life opens up possibilities for playing and imagination. It represents a transitional realm in which, according to Winnicott, the interplay between subjective experiences and external realities can foster new meanings and vitalization. Similarly, the introduction of an “artificial third” creates a digital environment in the nexus between the internal processes and technological tools. This space allows for unconstrained engagement with the elusive aspects of selfhood and the generation of alternative narratives. For example, the proof-of-concept in this article illustrates how the integration of AI-based externalization techniques made complex internal structures, such as polarized inner voices, more perceptible, and manageable. Engaging creatively with these externalized internal dynamics opened up new therapeutic avenues within the space created by the entrance of the artificial third.

However, the role of GenAI as an artificial third in therapy introduces challenges, primarily empathic failures (45). For example, in the current study, an empathic failure occurred when the AI's interpretations initially failed to resonate with the patient's inner experience, leading to frustration and a sense of alienation. The experience of a response (visually or textually) that feels familiar to the patient yet distant and alien resonates with Freud's concept of the uncanny (49), in which familiar things suddenly seem strange and unsettling, creating a mix of recognition and fear. This feeling of uncanny might be linked to the AI's interpretive processes, which at times may seem emotionally detached and could occasionally pose challenges for the patient. However, the experience of the uncanny can also arise from the way AI enables direct and vivid contact with internal projected contents, some of which may even be unconscious, and thus might simultaneously feel both familiar and distant from the patient. In light of these challenges, the ability to overcome empathic failures and become more experientially aligned with the patient, as the AI successfully managed to do in this study, holds significant therapeutic value in and of itself, as it represents the possibility of creating in the therapeutic space a path for healing and growth in places where there were previously experiences of early pain and their reenactments. Nevertheless, it is crucial to acknowledge that while GenAI can adapt and correct its responses, and despite creating an illusion of being another subject in the therapeutic space, it fundamentally cannot experience genuine human emotions or fully embody the depth of human empathy (23, 26, 50, 51).

In addition, the GenAI's ability, as demonstrated by DIVI, to offer constructive feedback at the end of the session might also make a unique contribution to the psychotherapeutic field. This functionality introduces a third interpretive voice into the therapeutic setting. Although this AI-generated perspective need not be accepted as an absolute truth or even as a subjective interpretation (23, 24), it is meaningful to augment mentalization abilities. It offers a novel vantage point for contemplating internal and interpersonal experiences, thereby enriching the therapeutic dialogue with a coherent and distinct layer of insight. The AI's suggestion for interpretation might become a shared reference point for both therapists and patients for further work, thereby enhancing the transparency of the therapeutic process for the patient and potentially also contributing to accelerating its deepening. Indeed, the presence of an additional artificial third agent within the therapeutic space, capable of offering ‘interpretations’—a capability previously exclusive to the therapist—fundamentally changes the nature of traditional therapeutic relationships and necessitates a re-evaluation of fundamental issues in therapeutic relations such as transference, countertransference, therapeutic alliance, etc (15, 27, 52). Thus, the therapist's experience with AI integration is critical (30). Concerns regarding the impact of AI on the therapist's role, expertise, and authority in the therapy space must be addressed.

### 4.1 Ethical considerations

Recent scholarly work emphasizes that, while GenAI chatbots may provide benefits for mental health support, they also raise significant ethical challenges that are often magnified when working with individuals experiencing mental health difficulties (53). Integrating GenAI into psychotherapy introduces a complex array of ethical considerations that demand deep and integrative understanding. Each aspect of these ethical concerns calls not only for acknowledgment, but also a thorough exploration and development of practical solutions and ethical guidelines (55, 56).

### 4.2 Data security and model training

The use of AI in therapy involves the processing of highly sensitive personal data, raising significant concerns regarding data security and privacy. Therefore, these tools should be used on the therapist's computer, as exemplified in the proof of concept above. In doing so, the patient's information would not be documented under their own user account, ensuring anonymity for the patient. Furthermore, adopting this approach means the therapist would be accountable and hold the obligation of instituting the needed protocols to safeguard the patient's data.

For example, if an AI system were trained on confidential therapy session transcripts, the risk would be twofold: the potential breach of data security and the ethical dilemma of using such intimate information for AI training. Patients must be fully informed about how their data will be used and must provide explicit consent. Even with consent, robust data protection measures are paramount to prevent unauthorized access and ensure the confidentiality of patient information. Therefore, apart from first name and sex, the therapist should carefully consider whether there is clinical value in the patient reporting additional information such as age and unique cultural aspects of these tools. To the extent that it is unnecessary, it is advisable to refrain from providing further details (17, 23, 53, 54).

### 4.3 Potential for misuse

The versatility of AI also opens the door to its potential misuse, both within and outside the therapeutic context. For instance, AI-generated insights or tools developed for therapy could be repurposed for commercial gain or other unintended purposes. Such repurposing could happen if a company decides to use therapeutic AI algorithms to analyze consumer behavior, thereby exploiting the intimate knowledge gained in a therapeutic setting for profit. Such scenarios highlight the need for strict regulations and ethical guidelines to prevent the exploitation of AI in ways that violate patients’ privacy and trust. Moreover, therapists must recognize that all AI-generated content from therapeutic sessions, including externalized internal voices and dialogue, are part of the patient's medical records. Therefore, therapists should refrain from engaging with or continuing conversations with the patient's externalized voices without their explicit consent, as this would constitute an unauthorized use of the patient's personal therapeutic material (18, 53, 55).

### 4.4 Inaccurate representation of patient experience

The accuracy with which AI represents a patient's inner experience is a critical ethical concern. For example, an AI model might generate an overly simplified or skewed depiction of a patient's emotional state, leading to misinterpretations by both the patient and therapist. This misrepresentation can have profound implications, possibly exacerbating the patients’ issues rather than alleviating them. Ensuring that AI systems are empathically attuned to and accurately reflect the complexity of human emotions is a significant ethical challenge. In this context, it is important to remember that if internal voices are externalized via “classic” therapeutic tools (e.g., patient drawings, psychodrama tools), the use of AI adds and “mixes” an additional external aspect of the patient's voice, which might be experienced by the patient as foreign, alien, or even frightening (28). Despite its attempts to appear empathic, GenAI fundamentally operates only as a statistical model and not as a real empathic subject (26). Consequently, GenAI systems inevitably function as distorted mirrors, offering a biased reflection of both the reality of the outside world and our inner experiences (57).

### 4.5 Epistemological authority

The introduction of AI into a therapeutic setting may inadvertently shift the source of epistemological authority from the therapist to the AI system. This shift can occur if a patient starts to place more trust in the AI's interpretation of the therapist's judgment. Such a situation could diminish the therapist's role and potentially undermine the therapeutic process, as the human element and the therapist's experiential knowledge are essential components of effective therapy (23, 50).

For instance, the proof of concept demonstrated in this paper brings to light an ethical concern regarding the summary and analysis of conversations by AI upon conclusion of its use, specifically in relation to assessing its potential impacts on the patient and the future direction of the psychotherapeutic process. This issue underscores the necessity of carefully evaluating the implications of AI interactions on patient outcomes and ongoing treatment approaches, highlighting the importance of ethical considerations in AI integration within clinical settings.

Specifically, the growing presence of AI in the therapeutic space raises critical concerns about the potential erosion of patient autonomy, agency and authentic self-expression. When AI systems become prominent interpreters of therapeutic dialogue, there is a risk that patients may feel their personal narrative and unique psychological experience becoming secondary to AI-mediated interpretations, potentially compromising the essential human-centered nature of psychotherapy. However, when thoughtfully integrated and maintained as a complementary tool, AI may also enhance patient agency by offering new pathways for self-reflection and engagement in the therapeutic process (7, 23, 58–60).

Therefore, despite the inherent epistemological bias in dialogue with artificial intelligence, it is imperative that therapists understand and clearly recognize that they bear exclusive responsibility for interpretation in therapy and the patient's mental state. Integrating artificial intelligence in therapy does not remove the therapist's responsibility. On the contrary, it increases the gravity of the therapist's responsibility (17, 32).

### 4.6 AI cultural and gender biases

Finally, the potential for bias in AI systems is an important ethical issue. AI models can inadvertently perpetuate societal and gender biases or stereotypes, leading to representations that do not align with an individual patient's reality. An example of this could be an AI system that interprets a patient's inner voice through the lens of cultural stereotypes, failing to reflect his authentic inner experience, and missing his unique cultural-based idioms of distress (61). These biases can be particularly problematic in mental health contexts, where AI systems trained on Western populations might misinterpret cultural expressions of emotional distress or reinforce gender-based assumptions regarding mental health presentations (28, 62).

As Vallor (57) emphasized in her influential work on AI ethics, these biases are further exacerbated by the digital divide, which systematically excludes certain population groups from digital spaces. This exclusion leads to the absence of their experience and needs in AI training datasets, perpetuating structural inequalities in AI systems. Consequently, it is crucial to use AI tools within the context of a therapeutic session, in which the therapist can serve as a mediator. The therapist's presence helps bridge the gap between the patient and AI, ensuring that the tool's outputs are interpreted and applied in a manner that respects the patient's individuality and cultural context.

This also emphasizes that, to address these cultural and gender biases, the development of AI systems in mental health requires a comprehensive framework that integrates diverse perspectives at every stage. This includes assembling diverse development teams and cultural consultants who can identify potential blind spots, utilizing training datasets that authentically represent various cultural expressions of mental health, and implementing systematic bias audits to evaluate AI outputs in different cultural contexts. Such measures, while requiring additional resources and time, are essential for creating AI systems that can effectively serve diverse populations while avoiding the perpetuation of existing healthcare disparities (28, 32, 57, 63).

Addressing these ethical challenges involves ongoing dialogue and collaboration among therapists, technologists, ethicists, and patients to develop guidelines and best practices that prioritize patient well-being, data security, and ethical use of technology in mental healthcare. This approach will ensure that AI serves as a valuable adjunct in therapy; optimally, it will enhance the therapeutic process, while at the same time upheld the highest ethical standards.

### 4.7 The SAFE-AI protocol: a clinical framework for AI integration in therapy

The theoretical and ethical considerations discussed above underscore the need for a structured implementation framework of AI-enhanced therapeutic techniques. **The SAFE-AI protocol** (Screening, Alignment, Facilitation, and Evaluation of AI-Enhanced Interventions) provides systematic guidelines for implementing AI-based externalization techniques while ensuring cultural sensitivity, gender representation, and therapeutic authenticity (28). While developed through our experience with VIVI and DIVI, the principles of the protocol apply broadly to various AI-enhanced therapeutic tools. Table 4 provides a detailed breakdown of this protocol, including general and specific clinical and ethical considerations, intervention guidelines, and a practical troubleshooting guide.

**Table 4:** SAFE-AI protocol for AI integration in psychotherapy.

Stage	General clinical & ethical considerations	Specific VIVI/DIVI tool considerations	Clinical interventions
S—Screening & **S**etup	• Therapist & patient readiness assessment (patient comfort, familiarity with technology, therapeutic needs) • Therapeutic space preparation • Informed consent • Cultural/gender sensitivity	• Internal dialogue capacity evaluation • Therapeutic alliance preparation including assessment of tool possible benefits and challenges • Inner-Voice representation consent	"Based on our work with your inner voices, I'd like to suggest using an AI tool to explore them further. Would you like to try exploring [specific voice] this way?” [Note for therapist: Emphasize potential AI challenges, including cultural and gender biases]
A—Alignment & Attunement	• Alliance monitoring • Triadic therapeutic space management • Ongoing consent verification • Power dynamics management	• Voice characterization alignment • AI representation accuracy • Active clinical presence	“Let's ensure the AI accurately represents your experience. How would you describe this voice's characteristics?”
F—Facilitation & Monitoring	• Therapeutic process management • Empathic attunement • Patient autonomy protection • Epistemological authority maintenance	• Real-time voice adjustment • Transference handling • Misrepresentation management • Therapeutic intervention modes	• Meta-observation: “I notice when this voice speaks, your body language changes..” • Direct intervention: “Could you share what fears underlie these judgments?” • Collaborative adjustment: “Should we adjust how the voice expresses itself?"
E—Evaluation & Integration	• Clinical documentation • Therapeutic integration assessment • Alliance evaluation • Session impact review	• Voice work integration • Mentalization • Representation accuracy review	“How was it to explore your inner voice this way? What felt helpful or challenging?”

The SAFE-AI protocol has emerged from our experience of implementing VIVI/DIVI tools in psychotherapy. It addresses the unique challenges of integrating AI into therapeutic settings while maintaining clinical integrity and ethical standards. This protocol specifically focuses on managing the triadic therapeutic space created by AI integration while preserving therapeutic alliance and ensuring patient safety.

^a^ Implementation: This protocol serves as a dynamic framework specifically designed for AI-enhanced externalization work, while addressing the unique challenges identified in our research. Each element should be adapted to individual clinical contexts, while maintaining core safety and ethical standards.

Screening and setup (pre-session): The screening phase requires a thorough assessment of patient readiness and preparation of the therapeutic environment for AI integration. The therapist evaluates the patient's capacity to engage with AI-mediated interventions while considering cultural and gender-specific factors. For externalization tools, such as VIVI and DIVI, assessment focuses on the patient's ability to work with externalized representations of internal experiences. Therapists establish clear therapeutic parameters and expectations to ensure an appropriate understanding of AI's role of AI in the therapeutic process.

Alignment and Attunement (Session Start): During the alignment phase, the therapist calibrates the AI representations to align them with therapeutic objectives while maintaining clinical authority. This involves guiding AI interactions, monitoring cultural and gender sensitivity in AI responses, and addressing patient comfort, familiarity with technology, and therapeutic needs. When implementing externalization tools, such as VIVI and DIVI, the therapist facilitates accurate characterization of the patient's internal experiences, ensuring authentic representation through both visual and dialogical elements. Throughout this process, the therapist actively verifies the patient's consent and engagement while maintaining professional oversight of the therapeutic direction.

Facilitation & Monitoring (During Session): Clinicians need to maintain vigilant oversight of empathic attunement and address any misalignments or empathic failures in AI interactions. When utilizing externalization tools such as VIVI and DIVI, the therapist must preserve the therapeutic alliance while managing potential cultural/gender disconnects in visual representations or dialogue-based interactions. Clinicians must also ensure that the AI-generated content remains therapeutically beneficial, maintaining clear therapeutic authority throughout the process.

Evaluation and Integration (Post-Session): The final phase focused on integrating the AI-enhanced therapeutic experience and planning future interventions. The therapist guides the reflection on the session's impact, documenting key interactions, and therapeutic progress. Special attention is given to how the AI-mediated externalization tools influence the patient's relationship with their internal experiences. This evaluation encompasses both immediate therapeutic gains and potential adjustments required for subsequent sessions.

Important Note on AI Implementation and Empathic Failures in SAFE-AI Protocol:

The SAFE-AI protocol provides structured guidance for integrating AI into therapy while maintaining clear therapeutic governance. While AI technology introduces valuable capabilities to therapeutic work, clinical responsibility and leadership remain firmly with therapists. The therapeutic relationship between clinicians and patients continues to be the foundational element, with the therapist directing and moderating AI's contribution of AI to the therapeutic process. This framework ensures that AI enhances rather than redirects therapeutic work with the therapist maintaining full professional authority and responsibility for clinical decisions and interventions.

A therapist's ability to maintain clinical authority is crucial when working with AI-generated empathic failures. Drawing on established literature on therapeutic empathic failures (45, 64–67), therapists must monitor continuously for misalignments, validate the patient's experience immediately when they occur, and use these moments as therapeutic material—as demonstrated in our proof-of-concept study when VIVI generated an image of Mira that felt disconnected from Meir's experience. Key strategies include pausing AI interaction to process the failure, making specific adjustments to AI prompts, and stopping AI use if needed. Table 5 provides comprehensive guidelines for managing empathic failures as well as other therapeutic challenges, such as alliance disruption, cultural representation issues, and technical-therapeutic balance in AI-enhanced therapy.

**Table 5:** Troubleshooting guide using DIVI and VIVI tools.

Challenge	Intervention strategy
Therapeutic alliance disruption	• Pause AI interaction immediately when alliance strain is detected • Explicitly address the impact on the therapeutic relationship • Validate any discomfort or concerns about AI integration • Reestablish direct therapeutic connection before continuing • Consider reducing or stopping AI tool usage if needed
Empathic failures	• Monitor continuously for misalignments in AI responses • Acknowledge and validate patient's experience immediately • Pause the interaction to process empathic failure • Use Empathic failure as therapeutic material to explore patient's reactions and inner world • Make specific adjustments to AI prompts and parameters • Consider the intensity and appropriateness of emotional content • Return to traditional dialogue if corrections are unsuccessful • Document successful correction strategies for future reference • Refer to relevant literature on empathic failures in traditional psychotherapy to guide the intervention and deepen the therapeutic process.
Cultural/gender representation	• Modify AI prompts to better reflect patient's cultural context • Adjust representation parameters based on patient feedback • Maintain ongoing dialogue about representation accuracy • Incorporate patient's preferred cultural metaphors and expressions • Consider alternative theraputic methods when needed
Technical-therapeutic balance	• Keep focus primarily on the therapeutic process, rather than on AI use • Use AI tools as supplements rather than central elements • Maintain therapeutic narrative through transitions • Integrate technical aspects naturally into therapeutic flow • Regular check-ins about tool effectiveness
Internal experience differentiation	• Clearly distinguish between AI representations and actual internal experiences • Use AI tools as exploratory aids rather than definitive representations • Help patient maintain healthy boundaries with AI-generated content • Regularly ground work in patient's authentic experience • Process any confusion between AI and actual internal voices

The following table outlines common challenges that may arise when using the DIVI and VIVI tools in therapy, along with the suggested intervention strategies.

^a^ Implementation: These strategies should be flexibly applied based on individual patient needs and therapeutic contexts. The focus should be on maintaining therapeutic effectiveness, while leveraging the benefits of AI tools. Therapists should document successful interventions and adapt strategies based on clinical experience and patient feedback.

### 4.8 Limitations and future research

Alongside the considerable potential of AI-based externalization tools in psychotherapy put forth in this theoretical study, integrating these technologies also entails meaningful challenges that warrant attention. First, there is the risk that the tools may generate inaccurate, harmful, or culturally biased content (28, 57). Second, gaps in resonance between the tool's output and the patient's inner experiences may emerge, demanding adept tuning of content by the therapist. Additionally, some patients may struggle to utilize such tools or place trust in them amid such intimate processes. Beyond these aspects, there is a danger that these tools may overshadow the therapist's role and undermine the bond of trust and intimacy between patient and therapist (27). Thus, AI integration requires abundant caution and awareness of these pitfalls to optimally contribute to the therapeutic process.

The primary limitation of this study is its theoretical nature: it presents an initial conceptual framework without extensive empirical validation. While this theoretical foundation is essential for mapping the clinical and ethical landscapes of AI integration in psychotherapy, further research is needed to fully evaluate the feasibility, efficacy, and potential challenges of these tools in clinical settings. A key limitation is the need to examine the performance of these tools across different patient demographics and comfort levels using technology. Additionally, while our proof of concept demonstrated basic feasibility, systematic investigation is required to understand how these tools function across various therapeutic approaches and clinical contexts.

Given the identified limitations and the preliminary nature of our findings, establishing a robust empirical validation is a critical next step. Accordingly, Building on insights from this conceptual work, we are now initiating a two-pronged research approach. The qualitative component examines therapeutic relationship dynamics, with particular attention to empathic failures and cultural biases in AI-enhanced therapy. The quantitative strand focuses on the feasibility of implementation and therapeutic outcomes. This comprehensive research strategy aimed to transform our theoretical foundations and the SAFE-AI protocol into evidence-based clinical practice. Our mixed-methods approach specifically addresses questions of generalizability raised by our initial theoretical framework, examining how factors such as patient characteristics, therapeutic setting, and clinician expertise influence the effectiveness of AI-enhanced externalization techniques. Future research should prioritize controlled trials to rigorously assess the effectiveness of these interventions with traditional externalization techniques that do not use GenAI while also examining their impact across diverse patient populations and therapeutic settings.

Although the SAFE-AI protocol was initially developed for our externalization tools, its principles broadly apply to active AI integration in therapeutic settings. Future research should evaluate both its specific application to externalization techniques and its broader utility as a framework for therapeutic AI implementation. This evaluation is crucial for establishing innovative approaches to validate the therapeutic practices.

## 5 Conclusions

In the current theoretical study, we explored the integration of a well-known therapeutic method of externalization with the advanced capabilities of GenAI. Specifically, as part of this study's proof of concept, two AI-based externalization tools–dialogic and visual–were developed and demonstrated within the psychotherapeutic space. This demonstration suggested potential value of using these techniques to enhance the psychological well-being of patients but also indicated risks and ethical considerations. These insights are exploratory and require further empirical validation.

This theoretical study represents an initial step toward the broader journey of incorporating AI into psychotherapy. Although the proof of concept in the current article is based on an AI-based externalization technique, it opens the door to numerous other potential AI applications and innovations in mental health care in general, and in the psychotherapeutic field in particular. As the field advances, more sophisticated and clinically validated AI tools are expected to emerge, further enhancing the therapeutic process and expanding the horizons of mental health treatments. The SAFE-AI protocol developed in this study provides a structured foundation for clinicians navigating this evolving landscape, while Table 5 presents a comprehensive troubleshooting guide for the DIVI and VIVI tools, with particular emphasis on managing empathic failures and maintaining therapeutic integrity and clinical responsibility.

Moreover, this theoretical study holds significant value in demonstrating the potential of clinicians to create psychotherapeutic tools with relative ease. This democratization of the creation of psycho-technological tools empowers clinicians to tailor highly technological therapeutic interventions to specific needs and contexts (32). Through active clinical involvement in AI tool development and implementation, we may reduce potential biases and shape AI technology to be more genuinely aligned with mental health promotion and therapeutic goals.

In conclusion, while this theoretical study offers an exploratory glimpse into the potential role of AI in psychotherapy, it also highlights the need for more rigorous empirical research to fully understand and harness the potential of AI in this field. Future research should aim to build on these preliminary insights, and systematically examine and validate AI-assisted therapeutic interventions to ensure their effectiveness, safety, and ethical integration.

## Data Availability

The original contributions presented in the study are included in the article/Supplementary Material, further inquiries can be directed to the corresponding author.

## References

[1] Holmes W Tuomi I. State of the art and practice in AI in education. Eur J Educ. (2022) 57(4):542–70. 10.1111/ejed.12533

[2] Omar M Levkovich I. Exploring the efficacy and potential of large language models for depression: a systematic review. J Affect Disord. (2025) 371:234–44. 10.1016/j.jad.2024.11.052 39581383

[3] Pinzolits R. (2024). AI in academia: an overview of selected tools and their areas of application. MAP Educ Human, 4, 37–50. 10.53880/2744-2373.2023.4.37

[4] Shaheen MY. Applications of artificial intelligence (AI) in healthcare: a review. Sci Open Prep. (2021). 10.14293/S2199-1006.1.SOR-.PPVRY8K.v1

[5] Van Heerden AC Pozuelo JR Kohrt BA. Global mental health services and the impact of artificial intelligence–powered large language models. JAMA Psychiatry. (2023) 80(7):662–4. 10.1001/jamapsychiatry.2023.1253 37195694

[6] Elyoseph Z Levkovich I. Beyond human expertise: the promise and limitations of ChatGPT in suicide risk assessment. Front Psychiatry. (2023) 14:1213141. 10.3389/fpsyt.2023.1213141 37593450 PMC10427505

[7] Denecke K. How do conversational agents in healthcare impact on patient agency? In: Proceedings of the 1st Worskhop on Towards Ethical and Inclusive Conversational AI: Language Attitudes, Linguistic Diversity, and Language Rights (TEICAI 2024). St Julians, Malta: Association for Computational Linguistics (2024). p. 1–8.

[8] Elyoseph Z Levkovich I. Comparing the perspectives of generative AI, mental health experts, and the general public on schizophrenia recovery: case vignette study. JMIR Ment Health. (2024) 11:e53043. 10.2196/53043 38533615 PMC11004608

[9] Elyoseph Z Levkovich I Shinan-Altman S. Assessing prognosis in depression: comparing perspectives of AI models, mental health professionals and the general public. Fam Med Community Health. (2024) 12(Suppl 1):e002583. 10.1136/fmch-2023-002583 38199604 PMC10806564

[10] Elyoseph Z Refoua E Asraf K Lvovsky M Shimoni Y Hadar-Shoval D. Capacity of generative AI to interpret human emotions from visual and textual data: pilot evaluation study. JMIR Ment Health. (2024) 11:e54369. 10.2196/54369 38319707 PMC10879976

[11] Hadar-Shoval D Elyoseph Z Lvovsky M. The plasticity of ChatGPT’s mentalizing abilities: personalization for personality structures. Front Psychiatry. (2023) 14:1234397. 10.3389/fpsyt.2023.1234397 37720897 PMC10503434

[12] Hadar-Shoval D Asraf K Mizrachi Y Haber Y Elyoseph Z. Assessing the alignment of large language models with human values for mental health integration: cross-sectional study using Schwartz’s theory of basic values. JMIR Ment Health. (2024) 11:e55988. 10.2196/55988 38593424 PMC11040439

[13] Levkovich I Elyoseph Z. Identifying depression and its determinants upon initiating treatment: chatGPT versus primary care physicians. Fam Med Community Health J. (2023) 11(4):e002391. 10.1136/fmch-2023-002391 PMC10582915 37844967

[14] Levkovich I Elyoseph Z. Suicide risk assessments through the eyes of ChatGPT-3.5 vs ChatGPT-4: vignette study. JMIR Ment Health. (2023) 10(10):e51232. 10.2196/51232 37728984 PMC10551796

[15] Parth K. Datz F. Seidman C. Löffler-Stastka H. (2017). Transference and countertransference: a review. Bull Menninger Clin, 81(2), 167–211. 10.1521/bumc.2017.81.2.167 28609147

[16] Shinan-Altman S Levkovich I Elyoseph Z. The impact of history of depression and access to weapons on suicide risk assessment: a comparison of ChatGPT-3.5 and ChatGPT-4. PeerJ. (2024) 12:e17468. 10.7717/peerj.17468 38827287 PMC11143969

[17] Haber Y Levkovich I Hadar-Shoval D Elyoseph Z. The artificial third: a broad view of the effects of introducing generative artificial intelligence on psychotherapy. JMIR Ment Health. (2024) 11:e54781. 10.2196/54781 38787297 PMC11137430

[18] Tal A Elyoseph Z Haber Y Angert T Gur T Simon T The artificial third: utilizing ChatGPT in mental health. Am J Bioeth. (2023) 23(10):74–7. 10.1080/15265161.2023.2250297 37812102

[19] Winnicott DW. Playing and Reality. London: Psychology Press (1991).

[20] Luis de Mello F Alves de Souza S. Psychotherapy and artificial intelligence: a proposal for alignment. Front Psychol. (2019). 10.3389/FPSYG.2019.00263 PMC6378280 30804863

[21] Gual-Montolio P Jaén I Martínez-Borba V Castilla D Suso-Ribera C. Using artificial intelligence to enhance ongoing psychological interventions for emotional problems in real- or close to real-time: a systematic review. Int J Environ Res Public Health. (2022) 19(13):7737. 10.3390/ijerph19137737 35805395 PMC9266240

[22] Horn RL Weisz JR. Can artificial intelligence improve psychotherapy research and practice? Adm Policy Ment Health. (2020) 47:852–5. 10.1007/s10488-020-01056-9 32715430

[23] Sedlakova J Trachsel M. Conversational artificial intelligence in psychotherapy: a new therapeutic tool or agent? Am J Bioeth. (2023) 23(5):4–13. 10.1080/15265161.2022.2048739 35362368

[24] Stade EC Stirman SW Ungar LH Boland CL Schwartz HA Yaden DB Large language models could change the future of behavioral healthcare: a proposal for responsible development and evaluation. npj Mental Health Research. (2024) 3:12. 10.1038/s44184-024-00056-z 38609507 PMC10987499

[25] Asman O Tal A Barilan YM. Conversational artificial intelligence—patient alliance turing test and the search for authenticity. Am J Bioeth. (2023) 23(5):62–4. 10.1080/15265161.2023.2191046 37130413

[26] Perry A. AI will never convey the essence of human empathy. Nat Human Behav. (2023) 7(11):1808–9. 10.1038/s41562-023-01675-w 37474839

[27] Holohan M Fiske A. “Like I’m talking to a real person”: exploring the meaning of transference for the use and design of AI-based applications in psychotherapy. Front Psychol. (2021) 12:720476. 10.3389/fpsyg.2021.720476 34646209 PMC8502869

[28] Timmons AC Duong JB Simo Fiallo N Lee T Vo HPQ Ahle MW A call to action on assessing and mitigating bias in artificial intelligence applications for mental health. Perspect Psychol Sci. (2023) 18(5):1062–96. 10.1177/17456916221134490 36490369 PMC10250563

[29] Calisto FM Nunes N Nascimento JC. Modeling adoption of intelligent agents in medical imaging. Int J Hum Comput Stud. (2022) 168:102922. 10.1016/j.ijhcs.2022.102922

[30] Calisto FM Fernandes J Morais M Santiago C Abrantes JM Nunes N Assertiveness-based agent communication for a personalized medicine on medical imaging diagnosis. Proceedings of the 2023 CHI Conference on Human Factors in Computing Systems (2023). p. 1–20

[31] Calisto FMGF. Towards the human-centered design of intelligent agents in medical imaging diagnosis (Doctoral dissertation). Lisbon: University of Lisbon (2024). 10.13140/RG.2.28353.33126)

[32] Elyoseph Z Gur T Haber Y Simon T Angert T Navon Y An ethical perspective on the democratization of mental health with generative AI. JMIR Ment Health. (2024) 11:e58011. 10.2196/58011 39417792 PMC11500620

[33] Vossen W Szymanski M Verbert K. The effect of personalizing a psychotherapy conversational agent on therapeutic bond and usage intentions. Proceedings of the 29th International Conference on Intelligent User Interfaces (2024). p. 761–71.

[34] White M. The externalizing of the problem and the re-authoring of lives and relationships. In: Selected Papers. Adelaide, New South Wales, Australia: Dulwich Centre (1989). p. 5–28.

[35] Banting R Lloyd S. A case study integrating CBT with narrative therapy externalizing techniques with a child with OCD: how to flush away the silly gremlin. A single-case experimental design. J Child Adolesc Psychiatr Nurs. (2017) 30(2):80–9. 10.1111/jcap.12173 28557252

[36] McGuinty E Armstrong D Carrière AM. A clinical treatment intervention for dysphoria: externalizing metaphors therapy. Clin Psychol Psychother. (2014) 21(5):381–93. 10.1002/cpp.1844 23686568

[37] Emunah R. Acting for Real: Drama Therapy Process, Technique, and Performance. 3rd ed. New York: Routledge (2019).

[38] Falconer CJ Davies EB Grist R Stallard P. Innovations in practice: avatar-based virtual reality in camps talking therapy: two exploratory case studies. Child Adolesc Ment Health. (2019) 24(3):283–7. 10.1111/camh.12326 32677219

[39] Hestbech AM. Reclaiming the inner child in cognitive-behavioral therapy: the complementary model of the personality. Am J Psychother. (2018) 71(1):21–7. 10.1176/appi.psychotherapy.20180008 29852754

[40] Khouri GS. Ego states through bipersonal psychodramatic experience: the externalization of internal roles (parts). Rev Brasil Psicodrama. (2022) 30(e0822):1–14. 10.1590/psicodrama.v30.538_IN

[41] White M Epston D. Narrative Means to Therapeutic Ends. New York: W.W. Norton (1990).

[42] Katz M Christensen MJ Vaz A Rousmaniere T. Cognitive role-playing techniques: Externalization of voices. In: Katz M Christensen MJ Vaz A Rousmaniere T, editors. Deliberate Practice of TEAM-CBT. SpringerBriefs in Psychology. Cham: Springer (2023). p. 95–99. 10.1007/978-3-031-46019-7_14

[43] Feniger-Schaal R Orkibi H. Integrative systematic review of drama therapy intervention research. Psychol Aesthet Creat Arts. (2020) 14(1):68–80. 10.1037/aca0000257

[44] Coaston SC. Taming the brain weasels: reducing self-criticism through externalization and compassion. J Creat Ment Health. (2020) 15(2):176–88. 10.1080/15401383.2019.1644695

[45] Haley J. The art of being a failure as a therapist. Am J Orthopsychiatry. (1969) 39(4):691–5. 10.1111/j.1939-0025.1969.tb02463.x 5803602

[46] Britton R. Subjectivity, objectivity, and triangular space. Psychoanal Quart. (2004) 73:47–61. 10.1002/j.2167-4086.2004.tb00152.x 14750465

[47] Freud S Breuer J Luckhurst N Bowlby R. Studies in Hysteria. (N. Luckhurst, Trans.). London: The Penguin Group (2004). (Original work published 1895).

[48] Ferrario A Sedlakova J Trachsel M. The role of humanization and robustness of large language models in conversational artificial intelligence for individuals with depression: a critical analysis. JMIR Ment Health. (2024) 11:e56569. 10.2196/56569 38958218 PMC11231450

[49] Freud S. The ‘uncanny’. In: Strachey J, editor & Translator. The Standard Edition of the Complete Psychological Works of Sigmund Freud, Vol. 17. London: Hogarth Press (1919) 17. p. 217–56.

[50] Ferrario A Facchini A Termine A. Experts or authorities? The strange case of the presumed epistemic superiority of artificial intelligence systems. Minds Machines. (2024) 34(3):30. 10.1007/s11023-024-09681-1

[51] Turkle S. Alone Together: Why we Expect More from Technology and Less from Each Other. New York: Basic Books (2011).

[52] Amirkhani F Norouzi Z Babaii S. Psychotherapist bots: transference and countertransference issues. In International Conference on Computer Ethics (2023) 1(1).

[53] Coghlan S Leins K Sheldrick S Cheong M Gooding P D’Alfonso S. To chat or bot to chat: ethical issues with using chatbots in mental health. Digital Health. (2023) 9. 10.1177/20552076231183542 37377565 PMC10291862

[54] Fiske A Henningsen P Buyx A. Your robot therapist will see you now: ethical implications of embodied artificial intelligence in psychiatry, psychology, and psychotherapy. J Med Internet Res. (2019) 21(5):e13216. 10.2196/13216 31094356 PMC6532335

[55] Elyoseph Z Hadar-Shoval D Asraf K Lvovsky M. ChatGPT outperforms humans in emotional awareness evaluations. Front Psychol. (2023) 14:1199058. 10.3389/fpsyg.2023.1199058 37303897 PMC10254409

[56] Vilaza GN McCashin D. Is the automation of digital mental health ethical? Applying an ethical framework to chatbots for cognitive behaviour therapy. Front Digit Health. (2021) 3:689736. 10.3389/fdgth.2021.689736 34713163 PMC8521996

[57] Vallor S. AI as Mirror: Artificial Intelligence and the Structure of the Self. Oxford: Oxford University Press (2024).

[58] Denecke K Abd-Alrazaq A Househ M. Artificial intelligence for chatbots in mental health: opportunities and challenges. Multiple Perspectives on Artificial Intelligence in Healthcare: Opportunities and Challenges. Cham, Switzerland: Springer (2021). p. 115–28.

[59] Khawaja Z Bélisle-Pipon JC. Your robot therapist is not your therapist: understanding the role of AI-powered mental health chatbots. Front Digit Health. (2023) 5:1278186. 10.3389/fdgth.2023.1278186 38026836 PMC10663264

[60] Sass R. Equity, autonomy, and the ethical risks and opportunities of generalist medical AI. AI Ethics. (2023):1–11. 10.1007/s43681-023-00380-8

[61] Nichter M. Idioms of distress revisited. Cult Med Psychiatry. (2010) 34:401–16. 10.1007/s11013-010-9179-6 20495999

[62] Levkovich I Shinan-Altman S Elyoseph Z. Can large language models be sensitive to culture in suicide risk assessment? J Cult Cogn Sci. (2024) 8(3):275–87. 10.1007/s41809-024-00151-9

[63] Tavory T. Regulating AI in mental health: ethics of care perspective. JMIR Ment Health. (2024) 11:e58493. 10.2196/58493 39298759 PMC11450345

[64] Kohut H. The Analysis of the Self: A Systematic Approach to the Psychoanalytic Treatment of Narcissistic Personality Disorders. Chicago, IL: University of Chicago Press (1971).

[65] Mordecai EM. A classification of empathic failures for psychotherapists and supervisors. Psychoanal Psychol. (1991) 8(3):251. 10.1037/h0079282

[66] Newhill CE Safran JD Muran JC. Negotiating the Therapeutic Alliance: A Relational Treatment Guide. New York, NY: Guilford Press (2003).

[67] Winnicott DW. The Maturational Processes and the Facilitating Environment: Studies in the Theory of Emotional Development. London: Routledge (1965).

